# Recent Advances in Heterologous Protein Expression and Natural Product Synthesis by *Aspergillus*

**DOI:** 10.3390/jof11070534

**Published:** 2025-07-17

**Authors:** Yuyang Sheng, Shangkun Qiu, Yaoming Deng, Bin Zeng

**Affiliations:** College of Pharmacy, Shenzhen Technology University, Shenzhen 518118, China

**Keywords:** *Aspergillus*, heterologous expression, natural products, *Aspergillus oryzae*, biosynthesis, terpenoid

## Abstract

The filamentous fungal genus *Aspergillus* represents an industrially significant group of eukaryotic microorganisms. For nearly a century, it has been widely utilized in the production of diverse high-value products, including organic acids, industrial enzymes, recombinant proteins, and various bioactive natural compounds. With the rapid advancement of synthetic biology, *Aspergillus* has been extensively exploited as a heterologous chassis for the production of heterologous proteins (e.g., sweet proteins and antibodies) and the synthesis of natural products (e.g., terpenoids and polyketides) due to its distinct advantages, such as superior protein secretion capacity, robust precursor supply, and efficient eukaryotic post-translational modifications. In this review, we provide a comprehensive summary of the advancements in the successful expression of heterologous proteins and the biosynthesis of natural products using *Aspergillus* platforms (including *Aspergillus niger*, *Aspergillus nidulans*, and *Aspergillus oryzae*) in recent years. Emphasis is placed on the applications of *A. oryzae* in the heterologous biosynthesis of terpenoids. More importantly, we thoroughly examine the current state of the art in utilizing CRISPR-Cas9 for genetic modifications in *A. oryzae* and *A. niger*. In addition, future perspectives on developing *Aspergillus* expression systems are discussed in this article, along with an exploration of their potential applications in natural product biosynthesis.

## 1. Introduction

Conventional microorganism chassis such as *Escherichia coli* and *Saccharomyces cerevisiae* face inherent constraints, including limited biosynthetic capacity, metabolic burden, and insufficient precursor supply, which collectively impair their ability to meet the escalating societal requirements for sustainable and eco-friendly chemical production [1,2,3]. As an essential group of industrial microorganisms in filamentous fungi, *Aspergillus* has been extensively utilized in various fields, including pharmaceuticals, food processing, agriculture, enzyme production, cosmetics, and chemical materials [4,5]. Over the past few years, the advancement of synthetic biology has further promoted the application of *Aspergillus* in the synthesis of critical pharmaceuticals and agrochemicals, such as penicillin/cephalosporin (antibiotics), statins (cholesterol control), echinocandin (antifungals), ergot alkaloids (α-blocking agent), citric acid (food additives), kojic acid (skin whitening), itaconic acid (antitumor), and gibberellin (as a plant growth stimulant) [6,7,8,9]. Compared to model organisms, such as *E. coli* and *S. cerevisiae*, *Aspergillus* species possess an expansive repertoire of secondary metabolic gene clusters, including polyketide synthases (PKS), non-ribosomal peptide synthetases (NRPS), and terpenoid synthases, enabling direct biosynthesis of complex natural products (e.g., lovastatin, penicillin) [10,11]. Moreover, *Aspergillus* provides an abundant supply of key precursors and coenzymes required for the biosynthesis of bioactive natural products, complemented by compatible transcription, translation, and post-translational modification machineries, including glycosylation, phosphorylation, and acetylation [12,13]. Thus, *Aspergillus* has become increasingly indispensable in synthetic biology research, especially as a heterologous host for proteins and natural products. For example, industrial fungal strains for organic acid production, such as *Aspergillus terreus* and *A. niger*, not only possess robust central carbon metabolism and highly efficient organic acid secretion capacity but also demonstrate excellent stress-resistant characteristics including acid tolerance and thermotolerance during industrial fermentation processes [14,15]. The edible strain *A. oryzae* has been successfully applied for the production of heterologous proteins, including bovine chymosin (CHY) and human lysozyme (HLY) [16].

Although many heterologous proteins have been successfully expressed in *Aspergillus* (e.g., human cytokine interleukin 6 (IL-6) in *A. niger* [17], adalimumab in *A. oryzae* [18]), there are still many challenges that need to be addressed, including improving production and optimizing expression systems. With the aid of *Aspergillus* expression platforms, the biosynthetic pathways of numerous natural products, including terpenoids, polyketides, and non-ribosomal peptides, have been elucidated [19]. For example, *A. nidulans* and *A. oryzae* have emerged as model organisms for filamentous fungal biology, which are currently recognized as preferred fungal chassis for heterologous production of bioactive natural products [19,20]. This has greatly facilitated the activation of silent gene clusters and the discovery of new compounds. Notably, among the *Aspergillus* expression platforms, *A. oryzae* exhibits exceptional advantages for terpenoid biosynthesis, making it an outstanding microbial host for the production of these compounds. For example, it has been efficiently engineered for the production of pleuromutilin (a diterpenoid antibiotic) and cephalosporin P1 (a triterpenoid antibiotic) [21,22].

In this review, we concentrate on the *A. niger*, *A. nidulans* and *A. oryzae* expression system and summarize recent advances in successfully expressing proteins and bioactive natural products through these species, emphasizing the biosynthesis of terpenoids through *A. oryzae*. Furthermore, we provide a comprehensive review of the latest advancements in the application of CRISPR-Cas9 for genetic engineering in *A. oryzae,* aiming to offer valuable insights for future exploration of *Aspergillus* chassis and the construction of high-efficiency fungal platforms.

## 2. *Aspergillus* Is an Expression Host for Heterologous Protein Production

### 2.1. A. niger

*A. niger*, as an indispensable industrial strain, has been extensively exploited for homologous and heterologous expressions of many protein products used in food, washing, textile, paper, and other industries [23] due to its high protein secretion efficiency, unique safety characteristics, and low–medium costs [24]. On the one hand, the genome of *A. niger* contains an extensive repertoire of genes encoding carbohydrate active enzymes (CAZymes) [25], making it an ideal host for CAZyme production and homologous expression. For example, Fiedler et al. used the tunable Tet-on system to control the *glaA* gene encoding glucoamylase (GlaA) and generated the Δ*racA* strain via *racA* gene deletion, leading to a 4-fold increase in GlaA secretion compared to the parental strain [26]. Moreover, Zhang et al. constructed gpdA promoters with one to four copies of the gpd box (PgpdA, PgpdA2B, PgpdA3B, PgpdA4B) and used them to regulate *xynB* expression in *A. niger.* The strain containing three copies of gpd box (PgpdA3B) demonstrated the highest xylanase activity (3588.38 U/mL), protein expression, and transcription efficiency [27]. Furthermore, Cai et al. cloned two β-glucosidase (BGL) genes from marine *A. niger ZJUBE-1* and then inserted them into the host genome for constitutive homologous expression. By leveraging the gpdA promoter to drive stable gene expression independent of inducers, they achieved directed expression of BGLs [28]. Similarly, Alazi et al. used the strong constitutive gpdA promoter to overexpress the transcription factor gaaR in *A. niger,* enabling constitutive transcription of pectinase-encoding genes, D-galacturonic acid (GA) transporter genes, and catabolic pathway enzymes even under non-inducing conditions [29]. Liu et al. cloned the tannase gene *tan7* from *A. niger SH-2* and overexpressed it in the low-background secretory strain *A. niger Bdel4* using the glaA promoter, achieving a peak tannase activity of 111.5 U/mL at 168 h with over 70% purity in the supernatant [30]. In addition, *A. niger* has successfully enabled the homologous expression of amylases, cellulases, and other CAZymes, facilitating their large-scale industrial production [31,32]. On the other hand, *A. niger* has also been used as a cell factory to express some heterologous proteins, including lysozyme [33], human lactoferrin [34], chymosin [35], thaumatin [36], lipase [37], and nuclease P1 [38]. However, achieving the efficient expression of most heterologous proteins is still difficult [39]. In recent years, driven by the rapid advancement and widespread application of gene editing technology, the CRISPR/Cas9 system has been effectively employed for genetic manipulation in *A. niger* [40]. For example, this technology enabled a significant enhancement in trehalase MthT production from *Thermothelomyces thermophilus*, with activity reaching up to 1698.83 U/mL [41]. Moreover, a CRISPR/Cas9-mediated multi-copy expression system for an alkaline serine protease from *A. oryzae* was successfully established in *A. niger*, yielding a protease activity of 11,023.2 U/mL and a protein concentration of 10.8 mg/mL [42].

### 2.2. A. oryzae

*A. oryzae* is identified as a GRAS (generally recognized as safe) organism by the U.S. Food and Drug Administration and has been used by the fermentation and food processing industries to produce sake, miso, soy sauce, and douchi for centuries [5]. Due to its strong protein secretion capabilities and reliable safety, *A. oryzae* is a suitable host for heterologous protein production [20]. Using *A. oryzae* as a host to express heterologous proteins has become increasingly well-established. For example, recombinant human lactoferrin [43], human lysozyme [44], calf chymosin [45], and other heterologous proteins have been successfully expressed to date. Moreover, in recent years, recombinant antibodies (such as adalimumab) with certain biological activity were also successfully expressed and purified in *A. oryzae* [18]. Additionally, cordycepin, a natural antibiotic with numerous pharmacological activities, was efficiently expressed by overexpressing two metabolic genes (*cns1* and *cns2*) involved in cordycepin biosynthesis [43].

### 2.3. A. nidulans

*A. nidulans* has long been recognized as a model organism for eukaryotic research, owing to its well-characterized genetics, physiology, and amenability to molecular manipulation [44,45]. Although *A. nidulans* is not a predominant industrial species, it serves as a promising microbial platform for industrial enzyme production. To date, *A. nidulans* has synthesized several industrial enzymes, including endoglucanase, xylanase, cellulases, β-glucosidases, laccases, and lipases [46]. Most of them were produced under submerged fermentation rather than solid-state fermentation due to the availability of fermentation parameters and reduced fermentation time of submerged fermentation [47]. In addition, *A. nidulans* is also a versatile fungal cell factory for the heterologous production of different carbohydrate active enzymes (CAZymes), which are industrially relevant biocatalysts responsible for the degradation of plant cell walls, with a promising result for the production of bio-based compounds from lignocellulosic feedstock, such as biofuel and nutraceuticals [48]. However, due to its relatively low protein secretion capacity, *A. nidulans* has not been fully developed as a cell factory for heterologous protein expression. Therefore, Yan et al. successfully developed a high-efficiency *A. nidulans* strain (*ΔagsB-derA*) by performing stepwise modification of mycelial morphology and protein secretory pathway to alleviate this limitation. Consequently, a higher yield of secretory and expression of human interleukin-6 (HuIL-6) was achieved by further disruption of extracellular proteases, indicating that *A. nidulans* is a promising platform for efficient heterologous protein expression [49]. Very recently, Gerhardt et al. employed CRISPR/Cas9 technology to examine how deleting genes related to N-glycan assembly and endoplasmic reticulum protein quality control affects recombinant β-xylosidase secretion in *A. nidulans*. Their findings demonstrated that combined *algC* and *algI* deletion enhances the secretion and alters the secretome, which may be a promising strategy for boosting recombinant protein secretion [50].

Although the application of heterologous protein expression using *A. niger*, *A. nidulans*, and *A. oryzae* as hosts has reached a relatively mature stage, in-depth studies on the regulation of gene expression and protein secretory pathways are still required to further optimize expression platforms, enhance protein yields, and ultimately establish highly efficient expression systems for heterologous proteins [20,51,52].

## 3. *Aspergillus* Is a Microbial Chassis for Natural Product Synthesis

In addition to being exploited as excellent hosts for heterologous protein expression, filamentous fungi have also been developed as ideal platforms for the production of natural products due to their exceptional capacity for secondary metabolite production, their inherent pre-mRNA splicing mechanisms, and their abundant supply of biosynthetic precursors and coenzymes [19,53]. Compared to typical heterologous hosts such as *E. coli* and yeast, the most notable advantage of filamentous fungi is their capability to express entire gene clusters responsible for the biosynthesis of fungal natural products [54,55]. Moreover, during the cloning of these gene clusters, there is no requirement to eliminate introns, as most filamentous fungal hosts can accurately execute the splicing of introns from secondary metabolite genes derived from other fungi, thereby successfully yielding the desired products [56,57]. Several filamentous fungi, such as *A. oryzae*, *A. nidulans*, and *A. niger*, have been successfully utilized for the study of biosynthetic pathways and heterologous production of natural products.

### 3.1. A. oryzae Is a Chassis for Natural Product Production

*A. oryzae* is a robust platform for the heterologous production of natural products, which are still abundant in modern drug discovery [58]. This platform not only facilitates the elucidation of the biosynthetic pathway of a given natural product but also allows the activation of silent or cryptic biosynthetic gene clusters, making a great contribution to simplifying the biosynthetic pathway, discovering new natural products and their derivatives, and genome mining. Thus, the *A. oryzae* host has been widely utilized for genome mining and the biosynthesis of fungal natural products, including polyketides, non-ribosomal peptides, and terpenoids (e.g., sesquiterpenoids, diterpenoids, sesterterpenoids) [59,60], which have always been considered as important ingredients in therapeutic agents.

#### 3.1.1. Transformation Methods in *A. oryzae*

**Figure 1:**
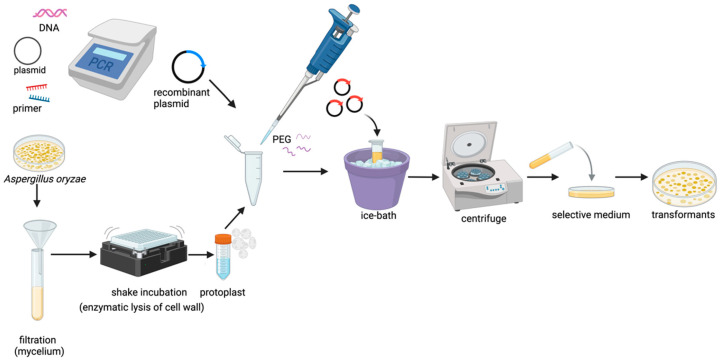
The process of protoplast-mediated transformation in *A. oryzae.* (Figure 1 was created with BioRender.com).

Currently, the heterologous expression of biosynthetic genes encoding natural products in *A. oryzae* primarily relies on the PEG/CaCl2-mediated protoplast transformation method [61], and the process is shown in Figure 1. Using molecular biology, the exogenous genes were loaded onto the vectors of nutrient-deficient screening markers and resistance markers, and the target gene vectors were transformed into protoplasts with the help of the high-sugar and -salt osmotic pressure of the transformation reagents. The target gene can be inserted into the chromosome of *A. oryzae* after homologous recombination between the vector and the host genome [62]. Transformed single genes or gene clusters can be inserted individually or as a whole. For example, Sakai et al. [58] successfully cloned the entire gene clusters (40 kb for monacolin K biosynthesis in *Monascus ruber* and 12 kb for terrequinone A biosynthesis in *A. nidulans*) into vectors via cosmid library construction, achieving heterologous expression of both gene clusters in the *A. oryzae* host. Single-gene insertion is a process in which the 5′ and 3′ ends of the target gene are added to an amylase (*amyB*) [63] or amylase-enhanced [64] promoter and terminator, respectively, and then induced by starch or dextrin in the culture medium, which enables the efficient expression of the target gene [21,65].

Although protoplast transformation enables efficient selection of transformants with successful target gene integration and facilitates isolation of high-expression strains, transgenerational instability in fungal transformants may result in either physical loss of the transgene or its transcriptional silencing [66]. Therefore, optimizing the *A. oryzae* expression system is significant for achieving high-efficiency transformation. For instance, Liu et al. and Wei et al. successively achieved targeted gene knock-in in *A. oryzae* using optimized CRISPR-Cas9 genome editing systems [67,68]. This approach enables precise genomic integration of foreign genes with high efficiency, significantly reducing screening time. Notably, the selectable marker can be automatically excised after integration, eliminating marker-associated limitations and facilitating sequential multigene insertions [67]. Recently, Li et al. improved the transformation and editing efficiency of the CRISPR/Cas9 system by optimizing the preparation conditions of protoplasts in *A. oryzae* [69].

#### 3.1.2. The Production of Terpenoids in *A. oryzae*

**Figure 2:**
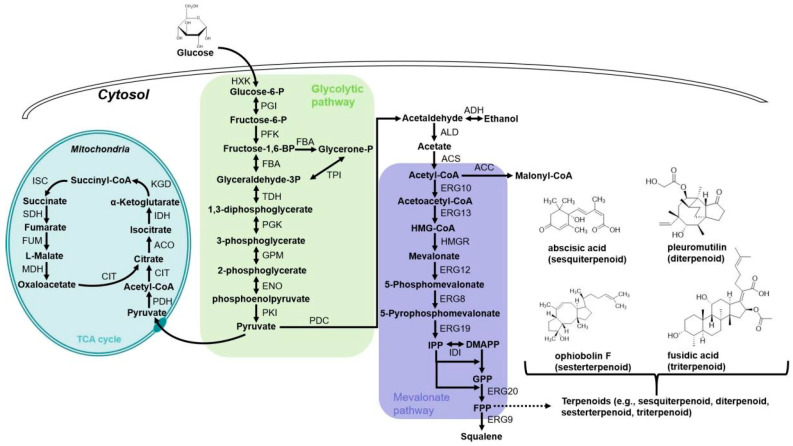
Metabolism pathway for terpenoid production in *A. oryzae*. HXK, hexokinase; PGI, phosphoglucose isomerase; PFK, phosphofructokinase; FBA, fructose-bisphosphate aldolase; TPI, triose phosphate isomerase; TDH, glyceraldehyde-3-phosphate dehydrogenase; PGK, phosphoglycerate kinase; GPM, phosphoglycerate mutase; ENO, enolase; PKI, pyruvate kinase; PDC, pyruvate decarboxylase; ADH, alcohol dehydrogenase; ALD, aldehyde dehydrogenase; ACS, acetyl-CoA synthase; ACC, acetyl-CoA carboxylase; ERG10, acetyl-CoA C-acetyltransferase; ERG13, hydroxymethylglutaryl-CoA synthase; HMGR, HMG-CoA reductase; ERG12, mevalonate kinase; ERG8, phosphomevalonate kinase; ERG19, diphosphomevalonate decarboxylase; IDI, Isopentenyl-diphosphate delta-isomerase; ERG20, FPP synthase; ERG9, squalene synthase; PDH, pyruvate dehydrogenase; CIT, citrate synthase; ACO, aconitase; IDH, isocitrate dehydrogenase; KGD, multifunctional 2-oxoglutarate metabolism enzyme; ISC, succinyl coenzyme A synthetase; SDH, succinatedehydrogenase; FUM, fumarase; MDH, malate dehydrogenase; IPP, isopentenyl pyrophosphate; DMAPP, dimethylallyl pyrophosphate; GPP, geranyl diphosphate; FPP, farnesyl pyrophosphate.

Terpenoids, the largest family and the most diverse group of natural products with over 90,000 structures reported, are derived from C_5_ of isopentenyl diphosphate (IPP) and its isomer dimethylallyl pyrophosphate (DMAPP). They are typically formed by terpene cyclases and further modified through oxidation, hydroxylation, glycosylation, or other enzymatic reactions [70]. According to the carbon numbers of the terpenoid molecule, they are categorized as monoterpenoids (C10), sesquiterpenoids (C15), diterpenoids (C20), sesterterpenoids (C25), or triterpenoids (C30) [71]. Due to their beneficial therapeutic properties including anti-cancer, anti-oxidation, antibacterial, antiviral, antimalarial, and potent anti-inflammatory activities, terpenoids are significant sources of natural product drugs, including menthol (monoterpenes), the well-known antimalarial drug artemisinin (sesquiterpenes), vitamin A, the anti-cancer drug paclitaxel (diterpenes), ginsenosides (triterpenoids), the cardiovascular drug tanshinone and so on [72,73,74,75,76]. In nature, terpenoid biosynthesis mainly occurs through two distinct pathways: the 2-C-methyl-D-erythritol-4-phosphate (MEP) pathway, predominantly in bacteria and plant plastids, and the mevalonate (MVA) pathway, primarily in eukaryotes and archaea [77]. As shown in Figure 2, *A. oryzae* possesses almost all precursor compounds for terpenoid synthesis, including IPP, DMAPP, geranyl pyrophosphate (GPP), farnesyl pyrophosphate (FPP), and geranylgeranyl pyrophosphate (GGPP) generated through the MVA pathway [78]. Moreover, *A. oryzae* exhibits robust protein secretion machinery and a superior post-translational modification capacity, enabling the precise recognition and splicing of introns. These attributes allow for the efficient heterologous expression of nearly all terpenoid cyclases in this fungal system [79]. Therefore, *A. oryzae* is an excellent heterologous expression chassis for terpenoids, particularly suitable for expressing fungal or plant-derived terpenoid biosynthetic gene(s)/clusters [80]. In recent years, *A. oryzae* has been widely applied in research on terpenoid biosynthesis, genomic mining, and synthetic biology. Here, we systematically review recent advances in terpenoid production using *A. oryzae* as a heterologous host, organized according to terpenoid classification systems. Table 1 presents the representative terpenoids heterologously produced in *A. oryzae*.

##### Sesquiterpenoids

Sesquiterpenoids are ubiquitously distributed in the tissues of plants, marine organisms, microorganisms, and insects. Although they comprise a conserved C_15_ carbon skeleton, sesquiterpenoids exhibit remarkable skeletal diversity through multienzymatic secondary metabolic processes, generating over 180 skeletal frameworks and complex derivatives [81,82]. Thus, sesquiterpenoids represent one of the largest and most structurally diverse classes of terpenoids in terms of both quantitative abundance and chemical complexity. Takino et al. identified a novel type of sesquiterpenoid synthase gene cluster from *Botrytis cinerea* and achieved the biosynthesis of abscisic acid using *A. oryzae* as a heterologous expression host [83]. Murai et al. utilized *A. oryzae* as a heterologous expression host to biosynthesize a sesquiterpene alcohol with a novel skeleton, trichobrasilenol [84]. Moreover, Feng et al. heterologously expressed braA (terpene cyclase), braB (N-acetylglucosaminidase), and braC (P450 oxidase) from *Annulohypoxylon truncatum* in *A. oryzae*, resulting in the biosynthesis of novel compounds brasilane A, brasilane D, and brasilane E [85]. Furthermore, Fukaya et al. applied the hot-spot knock-in method and incorporated 13 melleolide biosynthetic genes into *A. oryzae* through stepwise reconstitution. Consequently, they successfully isolated 1α-hydroxymelleolide, a representative sesquiterpenoid and natural melleolide derivative, and elucidated its biosynthetic pathway [86]. Additionally, using *A. oryzae* as a heterologous expression system, Han et al. characterized 12 putative sesquiterpene synthases and one P450 enzyme in basidiomycete *Flammulina velutipes*, ultimately leading to the biosynthesis of 16 distinct sesquiterpenoid compounds [87].

##### Diterpenoids

Diterpenoids, composed of four isoprene units, are a diverse class of natural products biosynthesized from the C20 precursor GGPP. They have high value in the pharmaceutical industry and exhibit diverse biological activities, including antitumor, anti-inflammatory, and immunosuppressive effects [88,89]. Fujii et al. successfully introduced the whole gene cluster into *A. oryzae* by using four different vectors and demonstrated the total biosynthesis of a typical diterpene aphidicolin [90]. Qin et al. identified an unusual chimeric terpene synthase (EvVS) by genome mining of the fungus *Emericella variecolor*. Through heterologous expression in *A. oryzae*, a novel tricyclic diterpene hydrocarbon, variediene, was successfully obtained [91]. Furthermore, Bailey et al. achieved the biosynthesis of the biologically active compound pleuromutilin using *A. oryzae* as a heterologous host [21]. In 2017, based on the previous research, Alberti et al. gradually expressed the biosynthetic gene of pleuromutilin in *A. oryzae* and finally revealed its complete biosynthetic pathway [92]. In recent years, employing heterologous hosts of *A. oryzae*, integrated with pathway engineering methodologies, researchers have obtained new pleuromutilin congeners with antimicrobial activities, which provide more options for the expansion of this family of drugs [93,94,95]. Notably, Saito et al. obtained an *A. oryzae* strain generated by engineering multiple metabolic pathways, containing 13 metabolic modifications, which showed excellent productivities of pleuromutilin (161.6 mg/L), an 8.5-fold increase compared to the control strains without any metabolic modifications [96]. Additionally, Tazawa et al. employed an *A. oryzae* expression system to reconstruct the biosynthetic pathway of brassicicenes, which are a series of diterpenes, and the generated transformant successfully biosynthesized novel brassicicene derivatives (brassicicene I) [97]. Moreover, by using a newly developed hot-spot knock-in and plasmid recycling genome editing method, Liu et al. reconstituted the biosynthetic gene cluster of erinacine (a bioactive diterpene) in *A. oryzae*, which generated erinacine Q and its intermediates [67]. Very recently, Xu et al. identified a novel biosynthetic gene cluster (*tdn*) by using a genome mining method in *Talaromyces adpressus* and achieved heterologous expression of *tdn* genes in *A. oryzae*. As a result, a new diterpenoid, cycloaraneosene-9-ol-8-one, and three known diterpenoids were determined [98].

##### Sesterterpenoids

Sesterterpenoids are a relatively small group among terpenoids and are predominantly synthesized through cyclization of the linear precursor geranylfarnesyl diphosphate (GFPP) [99]. Although sesterterpenoids are the rarest of all isoprenoids, they are widely distributed in higher plants, microorganisms, insects, and marine invertebrate organisms, especially sponges, and exhibit diverse biological properties including anti-cancer, anti-inflammatory, antiprotozoal, antitubercular, and antifeedant activities [100,101]. During a screening of putative diterpene synthase genes, Oikawa’s group identified the first sesterterpene synthase (AcOS) from *Aspergillus clavatus*, which simultaneously possesses the functions of class I sesterterpene synthase and prenyltransferase. They used the *A. oryzae* heterologous expression system and obtained sesterterpene ophiobolin F [102]. On this basis, Quan et al. discovered a new ophiobolin F synthase (AcldOS) in *Aspergillus calidoustus*, which is a homologous gene of AcOS. They achieved the heterologous expression of AcldOS in *A. oryzae* and elucidated the absolute structure of ophiobolin F and its potential biosynthesis pathway [103]. Similarly, Okada et al. identified and characterized the bifunctional sesterterpene synthase EvQS by the genome mining approach; they introduced it into *A. oryzae* for heterologous expression and successfully obtained quiannulatene and quiannulatic acid [99]. Additionally, Quan et al. achieved heterologous expression in *A. oryzae* of a small biosynthetic gene cluster from *Aspergillus calidoustus*. This gene cluster, composed of two genes, *acldAS* for a chimeric sesterterpene synthase and *acldA-P450* for a cytochrome P450 monooxygenase, enabled the biosynthesis of asperterpenol A and asperterpenol B [104]. Moreover, Qiao et al. discovered a unique sesterterpenoid gene cluster (consisting of *pstA* and *pstB)* and heterologously expressed both genes in *A. oryzae*, resulting in the production of two new sesterterpenoids, penisentenol and penisentone [105]. Notably, Liu’s group designed a further optimized *A. oryzae* chassis through an automated and high-throughput (auto-HTP) biofoundry workflow. Using this platform, they reconstituted 39 terpenoid BGCs into 208 *A. oryzae* strains and successfully elucidated the biosynthetic pathway for the sesterterpenoid mangicol J [80]. Recently, Yan et al. successfully achieved the heterologous production of bioactive fungal sesterterpenoids variecolin and variecolactone in *A. oryzae* by co-expressing the cytochrome P450 monooxygenase VrcB along with the sesterterpene synthase gene *vrcA*. Subsequently, they employed a series of *vrcB* homologues that were individually co-expressed with *vrcA* in the *A. oryzae* system, yielding three novel variecolin analogs [106].

##### Triterpenoids and Steroids

Triterpenoids possess versatile biological activities and are extensively utilized in the pharmaceutical, food, cosmetic, and chemical industries [107]. As a structurally diverse class of terpenoids, triterpenoids are ubiquitously distributed across natural sources and serve as a significant resource for drug discovery from natural products due to their anti-inflammatory, antibacterial, antiviral, anti-oxidant, anti-cardiovascular, anti-tumor, and immunomodulatory properties [108,109]. Lv et al. designated the *hel* gene cluster and individually introduced it into *A. oryzae*. Heterologous expression of this nine-gene cluster in *A. oryzae* enables the isolation of the fungi-derived triterpenoid antibiotic helvolic acid (~20 mg L^−1^) and the elucidation of its complete biosynthetic pathway [110]. Based on this, Cao et al. first identified the biosynthetic gene cluster of fusidic acid, which is the only fusidane-type antibiotic that has been clinically used. Consequently, they obtained fusidic acid and unraveled its full biosynthetic pathway through the introduction of the two uncharacterized genes (*fusC1* and *fusB1*) into the previously established six-gene-expression *A. oryzae* strain [111]. After that, the biosynthetic gene cluster of cephalosporin P1, which is also a fusidane-type antibiotic, was identified by the same group and stepwise reconstitution in *A. oryzae* enabled them to obtain cephalosporin P1 and characterize its complete biosynthetic pathway [22]. Similarly, Li et al. individually co-expressed the biosynthetic gene cluster of the antifungal fernane-type triterpenoid polytolypin in *A. oryzae*, leading to the elucidation of its biosynthetic pathway and the generation of 13 fernane-type triterpenoids [112]. Very recently, Cao et al. introduced *fsoA*, *fsoD*, *fsoE*, and *fsoF* individually into *A. oryzae* and demonstrated that this biosynthetic gene cluster comprising the four genes is sufficient for producing fuscoatroside, the first enfumafungin-type antibiotics that belong to triterpenoids [113]. Through biosynthetic reconstitution in *A. oryzae*, the complete biosynthetic pathway of fuscoatroside has been revealed, and 11 novel derivatives have finally been discovered.

**Table 1:** Heterologously produced representative terpenoids in *A. oryzae*, sources of the biosynthetic genes, and hosts used for the heterologous expression.

Natural Products	Structure Type	Gene Source	Heterologous Hosts	References
abscisic acid	sesquiterpenoid	*Botrytis cinerea SAS56*	*A. oryzae NSAR1*	[83]
trichobrasilenol	sesquiterpenoid	*T. atroviride FKI-3849*	*A. oryzae NSAR1*	[84]
brasilane A, D and E	sesquiterpenoid	*Annulohypoxylon truncatum CBS 140778*	*A. oryzae NSAR1*	[85]
1α-hydroxymelleolide	sesquiterpenoid	*Armillaria mellea FMC 543*	*A. oryzae NSPlD1*	[86]
pleuromutilin	diterpenoid	*Clitopilus passeckerianus ATCC 34646*	*A. oryzae NSAR1*	[21]
aphidicolin	diterpenoid	*Phoma betae PS-13*	*A. oryzae NSAR1*	[90]
brassicicene I	diterpenoid	*Pseudocercospora fijiensis 10CR-1-24*	*A. oryzae NSAR1*	[97]
erinacine Q	diterpenoid	*Hericiumerinaceusyamabushitake Y2*	*A. oryzae NSPlD1*	[67]
20-prenylpaxilline 22-prenylpaxilline	diterpenoid	*Tolypocladium inflatum*	*A. oryzae NSAR1*	[114]
cotylenin C, F, I and E	diterpenoid	*Talaromyces adpressus*	*A. oryzae NSAR1*	[115]
cycloaraneosene-9-ol-8-one	diterpenoid	*Talaromyces adpressus*	*A. oryzae NSAR1*	[98]
ophiobolin F	sesterterpenoid	*Aspergillus clavatus NRRL*	*A. oryzae NSAR1*	[102]
astellifadiene	sesterterpenoid	*Emericella variecolor NBRC32302*	*A. oryzae NSAR1*	[116]
quiannulatene quiannulatic acid	sesterterpenoid	*Emericella variecolor NBRC32302*	*A. oryzae NSAR1*	[99]
asperterpenoid A	sesterterpenoid	*Talaromyces wortmannii ATCC 26942*	*A. oryzae NSAR*	[117]
asperterpenol A asperterpenol B	sesterterpenoid	*Aspergillus calidoustus CBS121601*	*A. oryzae NSAR*	[104]
mangicol J	sesterterpenoid	*Fusarium graminearum J1-012*	*A. oryzae NSAR1*	[80]
variecolin	sesterterpenoid	*Aspergillus aculeatus ATCC 16872*	*A. oryzae NSAR1*	[106]
helvolic acid	triterpenoid	*Aspergillus fumigatus Af293*	*A. oryzae NSAR1*	[110]
fusidic acid	triterpenoid	*Acremonium fusidioides ATCC 14700*	*A. oryzae NSAR1*	[111]
cephalosporin P1	triterpenoid	*Acremonium chrysogenum ATCC 11550*	*A. oryzae NSAR1*	[22]
polytolypin	triterpenoid	*Polytolypa hystricis UAMH7299*	*A. oryzae NSAR1*	[112]
fuscoatroside	triterpenoid	*Humicola fuscoatra NRRL 22980*	*A. oryzae NSAR1*	[113]

#### 3.1.3. *A. oryzae* as a Chassis for Synthesis of Other Natural Products

##### Polyketides

Polyketides represent a structurally diverse class of natural products, characterized by their intricately assembled carbon skeletons derived from simple acyl building blocks [118]. More than 10,000 polyketide compounds have been discovered so far, among which clinically important representatives—including lovastatin (anti-cholesterol), erythromycin (antibiotic), azithromycin (antibiotic), and rapamycin (immunosuppressant)—serve as indispensable therapeutic agents in modern medicine [119,120]. With the development of synthetic biology and the rise of green manufacturing, an increasing number of researchers are employing *A. oryzae* as a chassis to modify and optimize the synthesis methods of polyketide compounds continuously. For example, He et al. heterologously expressed the citrinin biosynthetic genes in *A. oryzae*, with the non-reducing polyketide synthase gene (*pksCT*, annotated as *citS*) as the core component. Through heterologous co-expression of *citS* and downstream tailoring of enzyme genes (*citA–citE*) in *A. oryzae*, they achieved high-titer production of citrinin, validating its biosynthetic pathway and highlighting *A. oryzae* as a robust host for fungal polyketide biosynthesis [121]. Moreover, Sakai et al. successfully overexpressed the monacolin K (MK) gene cluster from *Monascus pilosus* in *A. oryzae*, resulting in the production of the corresponding metabolite, MK [58]. Furthermore, Yamamoto et al. reconstituted the biosynthetic gene cluster of phomoidride B in *A. oryzae* by using a genome editing-based hot-spot knock-in method, thereby elucidating the late-stage biosynthesis of phomoidrides [122]. Additionally, Han et al. identified a polyketide synthase (PKS, *herA*) in the basidiomycete *Hericium erinaceus* through genome mining. They cloned the *herA* gene and subsequently heterologously expressed it in *A. oryzae*, successfully achieving the production of orsellinic acid (OA) [123]. Similarly, Huang et al. identified the *Phy* biosynthetic gene cluster from *Aspergillus chevalieri BYST01*, which is responsible for the biosynthesis of physcion, a bioactive polyketide natural product derived from both plants and microorganisms. Through heterologous expression of key genes (*PhyF*, *PhyG*, and *PhyL*) in *A. oryzae*, they successfully reconstructed the biosynthetic pathway of physcion, thereby elucidating the molecular mechanisms underlying its production [124].

##### Non-Ribosomal Peptides

Non-ribosomal peptides (NRPs) represent a structurally diverse class of polypeptide secondary metabolites produced by various microorganisms. In contrast to polypeptides synthesized via ribosomal mechanisms, these compounds are assembled by multimodular enzyme complexes, such as non-ribosomal peptide synthetases (NRPSs) [125,126]. de Mattos-Shipley et al. identified the *Pscy* gene cluster from *Penicillium* species and heterologously expressed it in *A. oryzae.* This gene cluster, comprising a non-ribosomal peptide synthetase (NRPS) and a novel trans-N-methyltransferase (N-MeT), enabled the biosynthesis of cycloaspeptide A and cycloaspeptide E [127]. Moreover, Qi et al. identified a putative BGC (*cri*) from *Eurotium cristatum NWAFU-1*. They cloned the *criC* gene (encoding a non-ribosomal peptide synthetase, NRPS) and heterologously expressed it in *A. oryzae*, enabling the efficient production of the cyclic dipeptide compound cyclo-TA [128]. Recently, Katayama et al. utilized *A. oryzae* as a heterologous expression platform for cyclochlorotine, a fungal mycotoxin synthesized by NRPS. Through cyclochlorotine production in *A. oryzae*, they investigated the subcellular localization of five transmembrane proteins and confirmed the normal function of UstYa family proteins and transporters [129].

### 3.2. A. nidulans as a Chassis for Natural Product Synthesis

Similar to *A. oryzae*, *A. nidulans* has also been widely used in synthetic biology to study gene clusters from other species. As one of the most popular fungal hosts for the reconstitution of fungal biosynthesis pathways, *A. nidulans* has abundant secondary metabolite production capacity and an efficient gene targeting system with simple nutritional requirements and rapid growth and reproduction rates, so it is the most advanced and efficient molecular genetic system and a well-established model organism among filamentous fungi [57,130]. Furthermore, due to the successful expression of many types of natural products in *A. nidulans*, the application of *A. nidulans* as a versatile chassis has greatly facilitated the complete characterization and reconstruction of biosynthetic pathways for diverse natural products, as well as the discovery of new compounds [10,131]. In recent years, the *A. nidulans* expression system has successfully produced many fungal natural products. For example, by heterologously reconstructing the biosynthetic pathways in *A. nidulans*, the Chooi group successfully biosynthesized a set of natural products, including two polyketides ((M)-viriditoxin from *Paecilomyces variotii* [132] and stemphyloxin II from *Parastagonospora nodorum* [133]) and a benzazepine (alkaloid nanangelenin A from *Aspergillus nanangensis* [134]). Yuan et al. achieved the biosynthesis of cosmosporaside C, a fungal hybrid terpenoid saccharide, and elucidated the assembly line, which contained several unusual steps [135]; a group of dimeric indole piperazine alkaloids was also heterologously synthesized in *A. nidulans* by the combination of the non-ribosomal peptide synthetase (NRPS)-like gene *cpsA* and other genes, and one of them, campesine G, has been shown to have insecticidal activity [136]. Yang et al. identified a biosynthetic gene cluster (*bgt*) in the genome of *Trichoderma erinaceum F1-1*, a marine-derived fungus, which encodes the UbiA terpene synthase BgtA; then, they achieved the transcriptional activation of the silent *bgt* gene cluster through successful heterologous expression in *A. nidulans LO8030*, resulting in the isolation of eight previously unreported bergamotene-derived sesquiterpenoids [137].

### 3.3. A. niger Is a Chassis for Natural Product Synthesis

One of the earliest demonstrations of heterologous expression for a complete fungal biosynthetic pathway is the reconstitution of the BGC from *Penicillium chrysogenum* in *A. niger* [138]. Although it is not as widely utilized as *A. oryzae* and *A. nidulans* in the field of heterologous natural product biosynthesis, it is still a strong candidate host, especially for the production of non-ribosomal peptides (NRPs), due to its substantial industrial advantages [139]. For instance, Yeh et al. introduced *mica*, an NRPS-like gene from *A. nidulans* into *A. niger,* and their findings confirmed that only *micA* is necessary and sufficient for the synthesis of microperfuranone [140]. Meyer et al. successfully expressed the non-ribosomal peptide synthetase gene *esyn1*, derived from *Fusarium oxysporum*, in the Tet-on system of *A. niger*, yielding approximately 5 g/L of the cyclic depsipeptide enniatin [141]. In addition, the polyketide synthase gene *terA* from *A. terreus* was successfully expressed in *A. niger*, yielding a mixture of three distinct compounds with varying sizes and structures: orsellinic acid, 4-hydroxy-6-methylpyranone, and 6,7-dihydroxymellein [142]. Löhr et al. introduced the mushroom polyketide synthase genes from *Cortinarius odorifer CoPKS1–6* individually in *A. niger*; the result indicated that *CoPKS1* and *CoPKS4* are a new class of atrochrysone carboxylic acid synthases that either exclusively generate an octaketide or synthesize both hepta- and octaketides simultaneously [143].

## 4. Application of CRISPR/Cas9-Based Genome Editing Technology in *A. oryzae* and *A. niger*

The clustered regularly interspaced short palindromic repeats/CRISPR-associated protein 9 (CRISPR/Cas9) system is a highly efficient and revolutionary genome editing technology, originally derived from the adaptive immune system of bacteria and archaea [40]. This system is primarily composed of two core components: a single guide RNA (sgRNA) and the Cas9 nuclease. The sgRNA directs the system to target specific DNA sequences through complementary base pairing, while the Cas9 nuclease executes double-stranded DNA cleavage at the designated sites, enabling precise genome editing [40,144]. In recent years, CRISPR/Cas9-mediated genome editing technology has been effectively utilized in both fundamental research and industrial production of natural products and recombinant proteins in the genus *Aspergillus*, with *A. niger* and *A. oryzae* emerging as the most extensively utilized species [40,145,146].

### 4.1. CRISPR/Cas9-Based Genome Editing Technology for A. niger

*A. niger* possesses exceptional tolerance to extreme environmental conditions, high production economy, robust fermentation stability, and superior food safety; it has been granted generally recognized as safe (GRAS) status by the U.S. Food and Drug Administration (FDA) [32,147], so it is a suitable cell factory for diverse biotechnological applications [40]. In 2016, Kuivanen et al. first successfully established the CRISPR-Cas9 system for genetic engineering in *A. niger* [148]. To disrupt the catabolism of galactaric acid in *A. niger* and thereby generate an engineered strain capable of the efficiently producing galactaric acid, they identified seven genes—putatively encoding metabolic enzymes and transport proteins—through RNA sequencing, and then they deleted these genes by using CRISPR/Cas9 technology in conjunction with in vitro synthesized sgRNA. Subsequently, the same group successively achieved the effective deletion of *gluD* and *gluF* genes using the identical CRISPR/Cas9 strategy, which encode the NADPH-requiring 2-keto-L-gulonate reductase and NADPH-requiring 5-keto-d-gluconate reductase, respectively [149,150]. On this basis, Kuivanen et al. further developed an optimized CRISPR/Cas9 genome editing method for *A. niger,* based on in vitro-assembled Cas9/gRNA ribonucleoprotein (RNP) complexes, and achieved a 100% targeting efficiency for a single genomic target [151]. In terms of site-specific gene insertion, Sarkari et al. established an efficient genetic engineering toolbox for *A. niger*, which combines the GoldenMOCS cloning system (a Golden Gate-derived modular cloning strategy) with a CRISPR/Cas9-mediated integration approach to enable site-specific insertion of heterologous expression cassettes at the *pyrG* locus [152]. This system used a transiently expressed Cas9 (via a size-reduced AMA1 plasmid that is readily lost) and a split-*pyrG* marker for direct selection, achieving a high integration efficiency of up to 100%. Beyond targeted gene editing, transcriptional regulation of gene expression also serves as a pivotal approach for constructing and optimizing cell factories. The Tet-on system is an effective method for functional analysis of essential genes, allowing for precise modulation of gene expression levels in response to inducer concentrations [153,154]. In recent years, researchers have focused on integrating CRISPR/Cas9 genome editing technology with the Tet-on regulatory system in *A. niger*, aiming to quantitatively elucidate the impacts of differential expression levels of target genes on product biosynthesis. For example, Cairns et al. employed CRISPR-based genome editing to integrate the titratable Tet-on expression system upstream of *ageB*, *secG*, and *geaB* in *A. niger*. They utilized this strategy to investigate the functional associations between protein secretion, filamentous growth, and organic acid production [155]. Moreover, Zhang et al. replaced the native promoter of the *pyrG* gene in *A. niger* with a Tet-on inducible promoter through in situ promoter substitution mediated by CRISPR/Cas9 genome editing [156]. By titrating the concentration of the inducer doxycycline (Dox), they established a quantitative relationship between the differential expression levels of *pyrG* and the performance of citric acid fermentation, including titers and productivity. Very recently, a CRISPR/Cas9-based visual toolkit enabling multiplex integration at specific genomic loci has been established in *A. niger* [157], which allows simultaneous insertion of recombinant genes into multiple high-expression loci through the combined application of a visual multigene editing system (VMS) and the CRISPR-HDR system.

### 4.2. CRISPR/Cas9-Based Genome Editing Technology for A. oryzae

To achieve accurate and efficient genome editing in *A. oryzae* and better meet the demands of industrial production, the CRISPR-Cas9 system has been rapidly adopted in this filamentous fungus recently [144]. In 2016, Katayama et al. successfully established a CRISPR/Cas9-mediated genome editing method in *A. oryzae*, marking the first application of targeted mutagenesis in this filamentous fungus [146]. Using the CRISPR/Cas9 system, they knocked out the uracil synthesis gene *pyrG* (encoding orotidine 5′-phosphate decarboxylase) to construct a stable uridine/uracil auxotrophic *A. oryzae* strain, achieving a 10% mutagenesis efficiency with a characteristic 1 bp deletion at the target site 3–4 bp upstream of the PAM sequence. On this basis, the same group further applied this system in 2017 to delete the *ligD* gene—a DNA ligase gene participating in non-homologous end joining (NHEJ) in *A. oryzae* [158]. This study demonstrated that introducing the *ligD* mutation by genome editing is an effective strategy to improve gene targeting efficiency in *A. oryzae* industrial strains. Furthermore, Katayama et al. established an optimized CRISPR/Cas9 method using AMA1-based autonomous replicating genome editing plasmids harboring a drug resistance marker (*ptrA*) [159]. Notably, this system enabled highly efficient marker-free multiple gene modifications, with mutation efficiency significantly improved to 50–100% in both wild-type and industrial *A. oryzae* strains. Additionally, Li et al. developed a CRISPR/Cas9 genome editing method by optimizing protoplast preparation conditions, which further enhanced the transformation and multiplexed genome editing efficiency in *A. oryzae* (37.6% single-gene and 19.8% dual-gene editing efficiency) [69]. In this study, the morphological gene *yA* was identified as a promising selection marker for the rapid and precise isolation of positive transformants during genetic manipulation in *A. oryzae.* Very recently, a novel modular synthetic biology toolkit for *A. oryzae* was developed, featuring a recyclable ribonucleoprotein (RNP)-based CRISPR-Cas9 method [160]. Unlike traditional plasmid-based systems, this approach achieved gene editing through direct transformation of pre-assembled CRISPR-Cas9 RNP complexes, eliminating the need for plasmid-based encoding of Cas9 and sgRNAs. Notably, this RNP-based method demonstrates comparable efficiency and versatility to plasmid-based CRISPR-Cas9 systems for genetic engineering in *A. oryzae* [159].

## 5. Conclusions and Perspectives

With the rapid development of synthetic biology and molecular biology techniques, *Aspergillus* species exhibit substantial potential as expression hosts and play an increasingly vital role in the heterologous production of proteins and the synthesis of bioactive natural products. On the one hand, the engineered strains of *Aspergillus* enable the efficient production of heterologous proteins, significantly increasing their yields to meet industrial standards [24]. On the other hand, as an extraordinary chassis, *Aspergillus* can activate silent biosynthetic gene clusters (BGCs), facilitating the discovery of novel natural products, as well as promoting the elucidation of complete biosynthetic pathways, thus simplifying and optimizing their intricate synthesis routes [161]. For instance, *A. oryzae*, *A. niger,* and *A. nidulans* are being widely developed as research tools in synthetic biology [162,163]. Notably, *A. oryzae* serves as a powerful platform that not only enables rapid and efficient functional identification of natural product biosynthetic gene clusters but also facilitates genome-guided mining of bioactive natural products. Moreover, this system can be exploited for targeted engineering and optimization of functional genes to enhance natural product biosynthesis [58,80]. To date, *A. oryzae* has successfully enabled the heterologous expression of diverse natural product classes, particularly sesquiterpenoids, diterpenoids, and polyketides (PKs) [79,164,165]. Remarkably, this platform exhibits outstanding adaptability, efficiently integrating biosynthetic genes not only from filamentous fungi but also from basidiomycetes, bacteria, and even higher plants [166,167]. Such versatility has significantly accelerated the rapid elucidation and reconstruction of biosynthetic pathways for complex natural products, establishing *A. oryzae* as an ideal platform for synthetic biology research on natural products [5,62]. However, compared to *A. niger*, *A. oryzae* remains less amenable to complex genetic manipulation. The active non-homologous end-joining (NHEJ) pathway in *A. oryzae* inherently results in low homologous recombination rates, making targeted gene knockouts or integrations challenging [168,169]. Moreover, the rigid cell wall of *A. oryzae* significantly impairs the efficiency of conventional transformation approaches, including electroporation and biolistics [166]. Consequently, genetic manipulation of *A. oryzae* is predominantly dependent on polyethylene glycol (PEG)-mediated protoplast transformation, a laborious and time-consuming procedure that demands technical expertise [170]. In addition, despite the application of CRISPR/Cas9 technology in *A. oryzae*, its efficiency is constrained by multiple factors. The Cas9 protein exhibits low nuclear localization efficiency, and although partial improvement can be achieved through codon optimization and fusion with nuclear localization signals (NLS) as reported in previous studies [146], this remains a limiting factor. Furthermore, *A. oryzae* displays intrinsic resistance to most antifungal antibiotics. This characteristic leads to a scarcity of viable dominant selectable markers, compelling researchers to predominantly rely on auxotrophic markers such as pyrG and argB for genetic selection procedures [16,166].

Since 2015, the CRISPR/Cas system with its diverse engineering efficiencies has been progressively applied to genome editing in various filamentous fungi [171,172,173]; this system guides the Cas protein to target specific DNA sequences via the guide RNA (gRNA), enabling efficient and precise editing of the genome, including gene knockout, insertion, and point mutation, thereby contributing to the construction of a highly efficient filamentous fungal expression platform [174]. Although the CRISPR/Cas9 system has indeed enhanced the genome editing efficiency of *Aspergillus* species, it still faces several challenges. For example, in most non-model strains, the gene editing efficiency of the CRISPR/Cas system remains relatively low, which is determined by multiple factors (e.g., host cellular barriers and nucleic acid degradation mechanisms) [175]; another major limitation is unexpected off-target effects, which means the recognition sequence of the gRNA may bind to non-target DNA, resulting in non-specific editing and uncontrollable genomic variations [176]. In the future, the CRISPR/Cas9 system can be further optimized in multiple dimensions, including reducing the off-target effect, overcoming PAM sequence constraints of Cas proteins, and enhancing precise on-target editing efficiency [177].

This article provides a comprehensive overview of the latest research advances in the *Aspergillus* expression system for the heterologous expression of proteins and the biosynthesis of bioactive natural products, with a detailed focus on the progress in the heterologous biosynthesis of terpenoid compounds. Additionally, this article highlights the recent advancements in the application of CRISPR/Cas9-based genome editing technology in *A. oryzae* over the past few years. In the genus *Aspergillus*, *A. oryzae, A. nidulans* and *A. niger* are widely utilized for the efficient production of heterologous proteins and recognized as excellent chassis for the biosynthesis of natural products. These species have become a robust and versatile platform for synthetic biology research [62,80,178]. Heterologous expression hosts of the genus *Aspergillus* have achieved significant success in both heterologous protein expression and natural product biosynthesis. However, further refinement and optimization of this system remain imperative, including but not limited to developing novel transformation methods, enhancing transformation efficiency, identifying and designing more efficient enhanced promoters, and optimizing host metabolic pathways [170,179]. These optimization needs are not only critical for advancing fundamental research but also lay the groundwork for the broader application of *Aspergillus* in synthetic biology.

## Data Availability

No new data were created or analyzed in this study. Data sharing is not applicable to this article.
